# Impact of maternal organic food consumption and diet quality during pregnancy on offspring’s risk of inflammatory bowel disease: findings from a Danish National Birth Cohort Study

**DOI:** 10.3389/fnut.2025.1632729

**Published:** 2025-07-10

**Authors:** Olivia Mariella Anneberg, Sjurdur Frodi Olsen, Anne Vinkel Hansen, Anne Ahrendt Bjerregaard, Thorhallur Ingi Halldorsson, Tine Jess, Maiara Brusco De Freitas

**Affiliations:** ^1^Center for Molecular Prediction of Inflammatory Bowel Disease—PREDICT, Department of Clinical Medicine, Aalborg University, Copenhagen, Denmark; ^2^Department of Epidemiology Research, Statens Serum Institut, Copenhagen, Denmark; ^3^Department of Nutrition, Harvard T.H. Chan School of Public Health, Boston, MA, United States; ^4^Center for Clinical Research and Prevention, Copenhagen University Hospital—Bispebjerg and Frederiksberg, Copenhagen, Denmark; ^5^Faculty of Food Science and Nutrition, School of Health Sciences, University of Iceland, Reykjavik, Iceland; ^6^Department of Gastroenterology and Hepatology, Aalborg University Hospital, Aalborg, Denmark

**Keywords:** maternal diet, prenatal exposures, nutritional epidemiology, pediatric diseases, fetal programming, prenatal diet, longitudinal study

## Abstract

**Background:**

This study explores associations of maternal organic food consumption and diet quality during pregnancy with pediatric-onset inflammatory bowel disease (IBD) risk in offspring, including Crohn’s disease (CD) and ulcerative colitis (UC).

**Methods:**

Pregnant mothers and their offspring were enrolled in the Danish National Birth Cohort, a nationwide prospective cohort study, in 1996–2002. In gestational week 30, telephone interviews assessed overall maternal organic food consumption during pregnancy. In gestational week 25, a food frequency questionnaire assessed maternal diet during the previous 4 weeks, including six different organic food types (eggs, dairy, meat, fruit, vegetables, and cereals). A Healthy Eating Index evaluated maternal diet quality based on adherence to Danish official dietary guidelines. Offspring with pediatric-onset IBD (≤18 years) were identified in national patient registries. Cox regression explored associations of maternal organic food consumption and diet quality during pregnancy with offspring’s risk of pediatric-onset IBD, CD, and UC.

**Results:**

The study included 60,274 singleton mother–child pairs, of which 168 children developed pediatric-onset IBD. Frequent maternal organic food consumption during pregnancy was not significantly associated with offspring’s IBD risk (HR: 0.63; 95% CI: 0.33–1.19). However, frequent organic food consumption during pregnancy, particularly organic eggs and dairy, lowered offspring’s risk of CD (HR: 0.40; 95% CI: 0.17–0.94), but not UC (HR: 1.11; 95% CI: 0.41–3.00). Maternal diet quality during pregnancy was not significantly associated with offspring’s risk of IBD (HR: 0.99; 95% CI: 0.97–1.01), CD, and UC.

**Conclusion:**

In this large prospective cohort study, we show that maternal organic food consumption, particularly eggs and dairy, during pregnancy may lower offspring’s risk of pediatric-onset CD, but not UC.

## 1 Introduction

Inflammatory bowel disease (IBD) is an immune-mediated disease characterized by chronic intestinal inflammation. The disease, with its two subtypes Crohn’s disease (CD) and ulcerative colitis (UC), affects millions of people worldwide (1). Approximately 25% of patients are diagnosed in childhood, and Denmark is among the top three countries with the highest incidence of pediatric-onset IBD globally (2). The disease incidence increased drastically during recent decades (1, 2), particularly in newly industrialized areas that have adopted a Western lifestyle (1, 2). This indicates that IBD is caused not only by genetics, but also by environmental factors, such as urban living, pollution, antibiotic consumption, smoking, and physical activity, which may vary depending on the disease subtype (3, 4). Furthermore, maternal diet during pregnancy may influence IBD development in offspring, as suggested primarily by experimental studies with pregnant mice (5). In a Norwegian cohort study, Guo et al. (6) recently observed that a higher diet diversity, but not diet quality, in pregnancy decreased offspring’s risk of IBD, specifically UC. This has not been investigated or confirmed by others.

Organic foods are produced under regulations by the European Union with the aim of protecting the environment and biodiversity through limited use of matters such as pesticides, antibiotics, and synthetic food additives (7). Due to the reduced exposure to these components, frequent organic food consumption during pregnancy might protect the offspring against IBD. This is supported by animal studies, which demonstrated that prenatal exposure to pesticides like chlorpyrifos and nitenpyram induced gut microbiota dysbiosis and intestinal barrier damage, in both mothers and offspring (8, 9). Maternal consumption of synthetic food additives, such as the emulsifier polysorbate-80, had similar effects in offspring (10). Additionally, a meta-analysis of observational studies involving human participants identified an association between maternal antibiotic use during pregnancy and offspring’s long-term susceptibility to IBD (11). However, no studies have yet examined the link between maternal organic food consumption during pregnancy and the risk of IBD in offspring.

Consumers of organic foods are often motivated by their potential health benefits and tend to follow a healthier diet compared to the general population (12, 13). Given the interrelatedness between frequent organic food consumption and a healthy dietary pattern, it is imperative to analyze these dietary habits together to limit confounding. A Healthy Eating Index has been developed to measure the presumed healthiness of a person’s diet, using a scoring system that reflects the degree of adherence to country-specific official dietary guidelines (14). In Denmark, these guidelines consist of seven recommendations for achieving a high-quality diet to prevent nutritional deficiencies and lifestyle-related diseases (15). This is particularly important during pregnancy because of the crucial role of maternal nutrition in fetal growth and development (16).

Therefore, we aimed to use unique maternal dietary data from the Danish National Birth Cohort (DNBC) in combination with nationwide health data to investigate the association between organic food consumption during pregnancy and the risk of pediatric-onset IBD in offspring, taking diet quality into account using the Healthy Eating Index. We also analyzed this association for six different types of organic foods, including eggs, dairy, meat, fruits, vegetables, and cereals. Furthermore, as CD and UC have distinct etiologies, analyses were repeated where the two disease subtypes were studied separately.

## 2 Materials and methods

### 2.1 Study design and participants

The DNBC is a prospective nationwide cohort study of early life exposures and their long-term effects on disease susceptibility (17). General practitioners across Denmark recruited pregnant mothers between January 1996 and October 2002. Subsequently, mothers and their children were followed long-term, both in interviews, questionnaires, and by linkage with medical information from national patient registries.

All pregnant mothers speaking Danish and planning to carry to term were eligible for inclusion in the DNBC. The current study included all singleton mother–child pairs from the DNBC, where the mother responded to (1) the telephone interview in gestational week 30, including its single question about overall organic food consumption and (2) the food frequency questionnaire in gestational week 25 with plausible self-reported energy intakes (2,500–25,000 kJ/day), including its six questions about consumption of different organic food types. In addition, mother–child pairs were excluded if the child’s information was missing from national patient registries.

Children with pediatric-onset IBD were identified in Danish national patient registries and defined as ≥2 IBD hospital contacts within 2 years, consisting of inpatient contacts only or a combination of inpatient and outpatient contacts (18). Analyses were restricted to the children aged ≤18 years at the time of their first hospital contact. Another outcome of interest was the IBD subtype, determined using ICD-10 codes associated with the contacts. The disease subtype was classified as CD when both CD (ICD-10: DK50) and UC (ICD-10: DK51) were recorded.

### 2.2 Maternal dietary intake

Data collection is described elsewhere (17). In short, computer-assisted telephone interviews in gestational weeks 12 and 30, plus 6 and 18 months post-delivery, collected information on the characteristics of mothers and their children. The interview in gestational week 30 included a question about the maternal frequency of overall organic food consumption, with the four response options: “never,” “rarely,” “sometimes,” and “frequently.” Furthermore, a validated semi-quantitative food frequency questionnaire was mailed to the mothers in gestational week 25 to assess their consumption of >360 foods during the previous 4 weeks (17). The questionnaire also included questions about the frequency of organic food consumption, divided into the six food groups: eggs, dairy, meat, fruit, vegetables, and cereals, with the four response options: “never,” “sometimes,” “frequently,” and “always.” The variables were modeled as binary with the levels low and high frequency of consumption by combining responses “never” and “sometimes” as well as “frequently” and “always.”

Maternal diet quality was assessed with a Healthy Eating Index (14) using data on maternal food and nutrient intake during pregnancy, which were calculated from responses to the food frequency questionnaire as previously described (17). The Healthy Eating Index is a measure of the adherence to Danish official dietary guidelines (15, 19), ranging from 0 to 80, with higher scores reflecting a higher diet quality. The total index score is the sum of individual scores from 0 to 10 for eight dietary components, comprising three adequate (fruit and vegetables, dietary fiber, fish) and five moderation (meat, saturated fatty acids, sodium, soft drinks, added sugar) components. Higher scores were given with higher intakes of the adequate components and lower intakes of the moderation components. A description of scoring criteria used for each component is found in Supplementary Table 1.

### 2.3 Assessment of covariates

The telephone interviews collected information on maternal alcohol consumption during pregnancy (yes, no), maternal smoking during pregnancy (yes, no), maternal nutritional supplement use during pregnancy (yes, no), and breastfeeding duration (days). Maternal pre-pregnancy weight (kg) and height (cm) were assessed to calculate maternal pre-pregnancy body mass index (kg/m^2^). The interviews also assessed maternal occupation, which was classified according to the International Standard Classification of Education 2011 to determine maternal educational level (higher, medium, lower). International Standard Classification of Education 2011 levels 0–2 corresponded to lower education, 3–4 to medium education, and 5–8 to higher education (20). Information on maternal age at childbirth (years), maternal antibiotics use during pregnancy (0 courses, 1–2 courses, ≥3 courses), parental IBD diagnosis (yes, no), offspring’s sex (boy, girl), offspring’s antibiotics use during the first year of life (yes, no), and premature delivery (yes, no) was obtained from national patient registries. Maternal energy intake (MJ/day) was derived from nutrient calculations, as described elsewhere (13).

### 2.4 Statistical analysis

R was used for all statistical analyses at the significance level *α* = 0.05. Baseline characteristics were compared according to maternal organic food consumption during pregnancy using Kruskal-Wallis test for continuous variables and X^2^ test for categorical variables.

Pediatric-onset IBD in offspring was ascertained from the date of birth until the date of diagnosis, death, emigration, or 18th birthday, whichever occurred first. In primary analysis, we used the Cox proportional hazards model to explore the association between maternal overall organic food consumption during pregnancy and offspring’s risk of pediatric-onset IBD, using “never” as the reference. The variable was also modeled as a continuous variable to test for linear trend. In sensitivity analysis, we accounted for siblings within the cohort by only including the firstborn sibling in case the mother participated in the DNBC with more than one pregnancy.

Secondary analyses investigated the association between maternal diet quality during pregnancy and risk of pediatric-onset IBD in offspring, with the Healthy Eating Index modeled as a continuous variable. Furthermore, we also explored the association between maternal consumption of six different organic food groups (eggs, dairy, meat, fruit, vegetables, and cereals) and offspring’s risk of pediatric-onset IBD, using “low” consumption as the reference. Subgroup analysis explored associations for CD and UC separately. The proportional hazards assumption was tested for violation using the Schoenfeld residuals test, showing that none of the analyses violated the assumption (all *p* > 0.05).

Results were presented as hazard ratios (HR) with corresponding 95% confidence intervals (CIs) from unadjusted and adjusted analyses. All analyses were adjusted for the following covariates: maternal educational level, maternal pre-pregnancy body mass index, maternal smoking during pregnancy, maternal nutritional supplement use during pregnancy, maternal antibiotics use during pregnancy, and parental IBD diagnosis. Additionally, analyses of organic food consumption were further adjusted for diet quality. Covariates were selected *a priori* from the current knowledge about IBD risk factors and their associations with diet (3). Those with missing data on selected covariates were excluded, and their proportion was reported.

### 2.5 Ethical statement

The mothers who decided to participate in the DNBC gave written informed consent for themselves and their children. The DNBC was approved by the National Committee on Health Research Ethics in Denmark (ref. no. [KF] 01-471/94) and conducted according to the guidelines of the Declaration of Helsinki. Ethical approval is not required for registry-based studies in Denmark. The study followed the rules and regulations defined by the Danish Data Protection Agency.

## 3 Results

**Figure 1:**
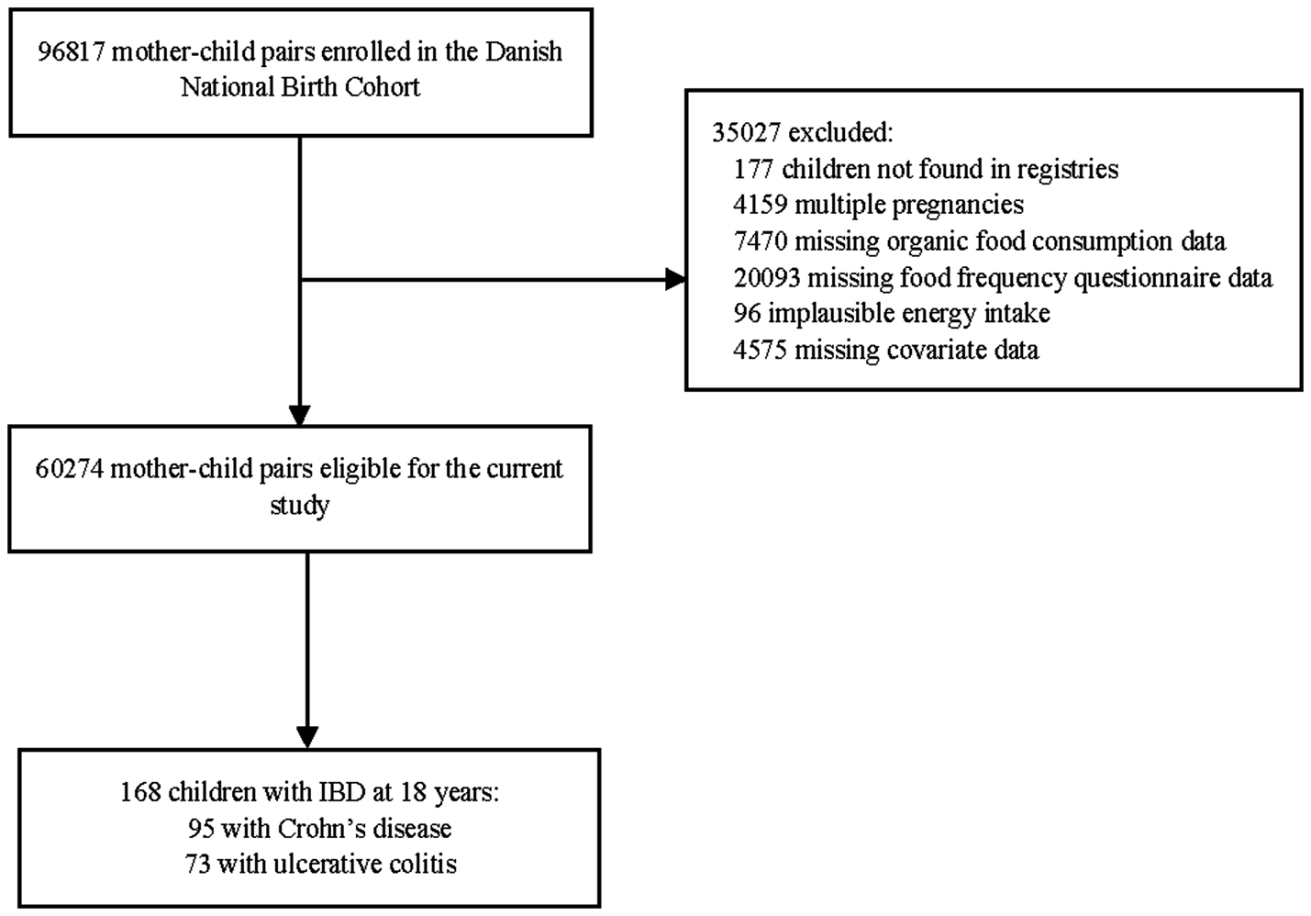
Flow chart of the study population. IBD, inflammatory bowel disease.

In total, 96,817 mother–child pairs were enrolled in the DNBC. After applying eligibility criteria, the current study consisted of 60,274 mother–child pairs (Figure 1). Supplementary Table 2 shows the baseline characteristics of the excluded compared to the included mother–child pairs. At the age of 18 years, 168 children had developed pediatric-onset IBD (0.28%) with a median (IQR) age at diagnosis of 15.0 (13.5–17.0) years (Supplementary Figure 1). The number of children with CD and UC, respectively, was 95 (0.16%) and 73 (0.12%).

Overall, the mothers had a median (IQR) age of 30.0 (27.0–33.0) years at birth, and 29,292 (48.6%) of the children were girls. The mothers had a median (IQR) Healthy Eating Index score of 22.2 (17.8–27.2) (Supplementary Figure 2). Individual scores for the eight components that comprise the Healthy Eating Index are presented in histograms in Supplementary Figure 3. The number of mothers with frequent organic food consumption was 13,742 (22.8%), while 22,841 (37.9%) mothers sometimes consumed them, 18,061 (30.0%) mothers rarely consumed them, and 5,603 (9.3%) mothers never consumed them. Mothers with frequent organic food consumption had higher diet quality compared to mothers with no consumption, as reflected by median (IQR) Healthy Eating Index scores of 25.4 (20.7–30.5) versus 19.6 (15.6–14.3). Furthermore, they had higher consumption of all organic food types (Table 1) and a higher daily energy intake, were older and more educated, had a lower pre-pregnancy body mass index, breastfed their children for longer, smoked less, drank less alcohol, consumed fewer antibiotics, and delivered preterm less often (Table 2).

**Table 1:** Maternal intake of different organic food groups according to overall organic food consumption during pregnancy.

Variable	Total (*n* = 60,274)	Never (*n* = 5,603)	Rarely (*n* = 18,061)	Sometimes (*n* = 22,841)	Frequently (*n* = 13,742)	*p*-value^1^
Organic egg consumption^2^						<0.001
Low, *n* (%)	32,833 (54.5%)	5,298 (94.6%)	14,596 (80.8%)	10,921 (47.8%)	2,018 (14.7%)	
High, *n* (%)	27,414 (45.5%)	305 (5.4%)	3,465 (19.2%)	11,920 (52.2%)	11,724 (85.3%)	
Organic dairy consumption^2^						<0.001
Low, *n* (%)	35,908 (59.6%)	5,509 (98.3%)	16,769 (92.8%)	12,260 (53.7%)	1,370 (10.0%)	
High, *n* (%)	24,339 (40.4%)	94 (1.7%)	1,292 (7.2%)	10,581 (46.3%)	12,372 (90.0%)	
Organic meat consumption^2^						<0.001
Low, *n* (%)	54,827 (91.0%)	5,557 (99.2%)	17,789 (98.5%)	21,525 (94.2%)	9,956 (72.4%)	
High, *n* (%)	5,420 (9.0%)	46 (0.8%)	272 (1.5%)	1,316 (5.8%)	3,786 (27.6%)	
Organic fruit consumption^2^						<0.001
Low, *n* (%)	51,986 (86.3%)	5,548 (99.0%)	17,610 (97.5%)	20,489 (89.7%)	8,339 (60.7%)	
High, *n* (%)	8,261 (13.7%)	55 (1.0%)	451 (2.5%)	2,352 (10.3%)	5,403 (39.3%)	
Organic vegetable consumption^2^						<0.001
Low, *n* (%)	45,830 (76.1%)	5,536 (98.8%)	17,354 (96.1%)	18,173 (79.6%)	4,767 (34.7%)	
High, *n* (%)	14,417 (23.9%)	67 (1.2%)	707 (3.9%)	4,668 (20.4%)	8,975 (65.3%)	
Organic cereal consumption^2^						<0.001
Low, *n* (%)	44,642 (74.1%)	5,553 (99.1%)	17,497 (96.9%)	17,657 (77.3%)	3,935 (28.6%)	
High, *n* (%)	15,605 (25.9%)	50 (0.9%)	564 (3.1%)	5,184 (22.7%)	9,807 (71.4%)	

^1^Tested with X^2^ test. ^2^Intakes of organic food types were modeled as a binary variable (low vs. high frequency of consumption) by combining responses “never” and “sometimes” as well as “frequently” and “always,” respectively.

**Table 2:** Baseline characteristics according to maternal organic food consumption during pregnancy.

Variable	Total (*n* = 60,274)	Never (*n* = 5,603)	Rarely (*n* = 18,061)	Sometimes (*n* = 22,841)	Frequently (*n* = 13,742)	*p*-value^1^
Maternal diet quality						<0.001
Median (IQR)	22.2 (17.8–27.2)	19.6 (15.6–14.3)	20.4 (16.3–24.9)	22.7 (18.4–27.3)	25.4 (20.7–30.5)	
Maternal age at birth (years)						<0.001
Median (IQR)	30.0 (27.0–33.0)	28.0 (26.0–31.0)	29.0 (26.0–32.0)	30.0 (27.0–33.0)	31.0 (28.0–34.0)	
Maternal educational level						<0.001
Lower education, *n* (%)	7,325 (12.2%)	1,355 (24.2%)	2,617 (14.5%)	2,210 (9.7%)	1,143 (8.3%)	
Medium education, *n* (%)	23,654 (39.3%)	2,746 (49.0%)	8,282 (45.9%)	8,523 (37.3%)	4,103 (29.9%)	
Higher education, *n* (%)	29,268 (48.6%)	1,502 (26.8%)	7,162 (39.7%)	12,108 (53.0%)	8,496 (61.8%)	
Maternal pre-pregnancy body mass index (kg/m^2^)						<0.001
Median (IQR)	22.6 (20.7–25.4)	23.5 (21.2–26.8)	23.1 (21.0–26.0)	22.5 (20.6–25.1)	22.0 (20.3–24.3)	
Maternal smoking						<0.001
No, *n* (%)	50,515 (83.8%)	4,293 (76.6%)	14,639 (81.1%)	19,444 (85.1%)	12,139 (88.3%)	
Yes, *n* (%)	9,732 (16.2%)	1,310 (23.4%)	3,422 (18.9%)	3,397 (14.9%)	1,603 (11.7%)	
Maternal nutritional supplement use						<0.001
No, *n* (%)	1,276 (2.1%)	187 (3.3%)	364 (2.0%)	414 (1.8%)	311 (2.3%)	
Yes, *n* (%)	58,971 (97.9%)	5,416 (96.7%)	17,697 (98.0%)	22,427 (98.2%)	13,431 (97.7%)	
Maternal alcohol intake						<0.001
No, *n* (%)	29,790 (49.4%)	3,300 (58.9%)	9,452 (52.3%)	10,718 (46.9%)	6,320 (46.0%)	
Yes, *n* (%)	30,457 (50.6%)	2,303 (41.1%)	8,609 (47.7%)	12,123 (53.1%)	7,422 (54.0%)	
Maternal antibiotics use						<0.001
0 courses, *n* (%)	42,239 (70.1%)	3,751 (66.9%)	12,506 (69.2%)	16,190 (70.9%)	9,792 (71.3%)	
1–2 courses, *n* (%)	14,063 (23.3%)	1,461 (26.1%)	4,390 (24.3%)	5,166 (22.6%)	3,046 (22.2%)	
≥3 courses, *n* (%)	3,945 (6.5%)	391 (7.0%)	1,165 (6.5%)	1,485 (6.5%)	904 (6.6%)	
Parental IBD						0.090
No, *n* (%)	59,549 (98.8%)	5,555 (99.1%)	17,835 (98.7%)	22,568 (98.8%)	13,591 (98.9%)	
Yes, *n* (%)	698 (1.2%)	48 (0.9%)	226 (1.3%)	273 (1.2%)	151 (1.1%)	
Maternal energy intake (MJ/day)						<0.001
Median (IQR)	9.8 (8.3–11.6)	9.9 (8.2–11.9)	9.7 (8.1–11.5)	9.8 (8.3–11.5)	9.9 (8.4–11.7)	
Missing data	2,331 (3.9%)	176 (3.1%)	660 (3.7%)	924 (4.0%)	571 (4.2%)	
Offspring sex						0.211
Girl, *n* (%)	29,292 (48.6%)	2,660 (47.5%)	8,747 (48.4%)	11,190 (49.0%)	6,695 (48.7%)	
Boy, *n* (%)	30,955 (51.4%)	2,943 (52.5%)	9,314 (51.6%)	11,651 (51.0%)	7,047 (51.3%)	
Offspring antibiotics use						<0.001
0 courses, *n* (%)	35,490 (58.9%)	3,100 (55.3%)	10,044 (55.6%)	13,568 (59.4%)	8,778 (63.9%)	
≥1 courses, *n* (%)	24,757 (41.1%)	2,503 (44.7%)	8,017 (44.4%)	9,273 (40.6%)	4,964 (36.1%)	
Breastfeeding duration^2^ (days)						<0.001
Median (IQR)	180 (134–180)	180 (90–180)	180 (120–180)	180 (150–180)	180 (180–180)	
Missing data, *n* (%)	11,737 (19.5%)	1,061 (18.9%)	3,328 (18.4%)	4,476 (19.6%)	2,872 (20.9%)	
Preterm delivery						0.004
No (≥37 weeks), *n* (%)	57,294 (95.1%)	5,326 (95.1%)	17,213 (95.3%)	21,778 (95.3%)	12,977 (94.4%)	
Yes (<37 weeks), *n* (%)	2,179 (3.6%)	225 (4.0%)	710 (3.9%)	805 (3.5%)	439 (3.2%)	
Missing data, *n* (%)	774 (1.3%)	52 (0.9%)	138 (0.8%)	258 (1.1%)	326 (2.4%)	

^1^Categorical variables were tested with X^2^ test, and continuous variables were tested with Kruskal-Wallis test. ^2^Truncated at 180 days. IBD, inflammatory bowel disease.

Frequent maternal organic food consumption during pregnancy, compared to never, did not significantly protect offspring against pediatric-onset IBD (adjusted HR: 0.63; 95% CI: 0.33–1.19; p_trend_ = 0.167). However, it was associated with a 60% lower CD risk in offspring (adjusted HR: 0.40; 95% CI: 0.17–0.94; p_trend_ = 0.030), whereas no association was found with offspring’s UC risk (adjusted HR: 1.11; 95% CI: 0.41–3.00; p_trend_ = 0.705) (Table 3). The results were unchanged when analyses were restricted to firstborn children in cases where the mother had participated in the DNBC with more than one pregnancy (Supplementary Table 3). Moreover, higher maternal diet quality during pregnancy was not associated with offspring’s risk of pediatric-onset IBD (adjusted HR: 0.99; 95% CI: 0.97–1.01), CD, or UC (Table 4).

**Table 3:** Association between maternal organic food consumption during pregnancy and the risk of pediatric-onset inflammatory bowel disease in offspring.

Outcome	Never (*n* = 5,603)	Rarely (*n* = 18,061)	Sometimes (*n* = 22,841)	Frequently (*n* = 13,742)	p_trend_^3^
Inflammatory bowel disease					
Cases, *n* (%)	17 (0.30%)	55 (0.30%)	71 (0.31%)	25 (0.18%)	
Unadjusted HR (95% CI)^1^	Reference	1.01 (0.59–1.74)	1.04 (0.62–1.77)	0.62 (0.34–1.15)	0.109
Adjusted HR (95% CI)^2^	Reference	0.98 (0.57–1.70)	1.03 (0.60–1.78)	0.63 (0.33–1.19)	0.167
Crohn’s disease					
Cases, *n* (%)	11 (0.20%)	34 (0.19%)	39 (0.17%)	11 (0.08%)	
Unadjusted HR (95% CI)^1^	Reference	0.97 (0.50–1.91)	0.89 (0.45–1.73)	0.42 (0.18–0.98)	0.029
Adjusted HR (95% CI)^2^	Reference	0.89 (0.45–1.76)	0.81 (0.41–1.61)	0.40 (0.17–0.94)	0.030
Ulcerative colitis					
Cases, *n* (%)	6 (0.11%)	21 (0.12%)	32 (0.14%)	14 (0.10%)	
Unadjusted HR (95% CI)^1^	Reference	1.10 (0.44–2.71)	1.33 (0.56–3.19)	0.99 (0.38–2.57)	0.942
Adjusted HR (95% CI)^2^	Reference	1.14 (0.46–2.83)	1.46 (0.60–3.55)	1.11 (0.41–3.00)	0.705

^1^Cox proportional hazards model; ^2^Cox proportional hazards model adjusted for maternal educational level, diet quality during pregnancy, pre-pregnancy body mass index, smoking during pregnancy, nutritional supplement use during pregnancy, antibiotics use during pregnancy, and parental inflammatory bowel disease diagnosis. ^3^Test for linear trend by modeling maternal organic food consumption as a continuous variable. CI, confidence interval; HR, hazard ratio.

**Table 4:** Association between maternal diet quality during pregnancy and the risk of pediatric-onset inflammatory bowel disease in offspring.

Outcome	Cases, *n* (%)	Unadjusted HR (95% CI)^1^	Adjusted HR (95% CI)^2^
Inflammatory bowel disease	168 (0.28%)	0.99 (0.97–1.01)	0.99 (0.97–1.01)
Crohn’s disease	95 (0.16%)	0.98 (0.95–1.01)	0.98 (0.95–1.01)
Ulcerative colitis	73 (0.12%)	1.00 (0.97–1.03)	1.01 (0.97–1.04)

^1^Cox proportional hazards model; ^2^Cox proportional hazards model adjusted for maternal educational level, pre-pregnancy body mass index, smoking during pregnancy, nutritional supplement use during pregnancy, antibiotics use during pregnancy, and parental inflammatory bowel disease diagnosis. CI, confidence interval; HR, hazard ratio.

Among the six organic food types, high compared to low consumption of organic eggs during pregnancy was associated with a 30% lower IBD risk in offspring (adjusted HR: 0.70, 95% CI: 0.51–0.95). This reduction was specifically attributed to a 41% lower risk of CD (adjusted HR: 0.58; 95% CI: 0.37–0.90) but not UC (adjusted HR: 0.88; 95% CI: 0.55–1.43) (Supplementary Table 4). Similarly, high compared to low consumption of organic dairy was associated with a 44% lower CD risk in offspring (adjusted HR: 0.56; 95% CI: 0.35–0.90), whereas no association was found with UC risk (adjusted HR: 1.15; 95% CI: 0.70–1.88) (Supplementary Table 5). These associations remained significant after adjusting for potential confounders, including maternal diet quality during pregnancy. In contrast, no associations were found for maternal high compared to low consumption of organic meat, fruit, vegetables, or cereals (Supplementary Tables 6–9).

## 4 Discussion

This nationwide prospective cohort study with more than 60,000 mother–child pairs from Denmark provides novel insights into the influence of maternal organic food consumption during pregnancy on the offspring’s development of pediatric-onset IBD. Although frequent organic food consumption did not protect the offspring against IBD, we found that it did protect against CD specifically, but not UC. Among the different organic food types, only eggs and dairy were associated with a lower CD risk in offspring, whereas consumption of organic meat, fruit, vegetables, and cereals was not associated with offspring’s disease risk. Likewise, higher diet quality was also unrelated to the offspring’s risk of IBD, CD, and UC.

Our findings suggest that frequent maternal organic food consumption during pregnancy, particularly organic eggs and dairy, may protect offspring against CD development during childhood. This discrepancy between CD and UC may reflect differences in their underlying pathogenesis and etiology, such as gut microbiota dysbiosis, which is often more severe with CD than UC (21). Pesticide residues have consistently been linked to microbiota dysbiosis (22), and organic food consumption may help reduce exposure to these potentially harmful compounds, as their concentrations are approximately five times lower in organic foods compared to conventional alternatives (23). This may benefit not only the mother but also the offspring, given that the maternal gut microbiota influences the offspring’s gut microbiota maturation (24, 25) and the in utero immune environment (26). Nevertheless, the absent associations for maternal consumption of plant-based organic foods imply a role of organic production-specific characteristics beyond pesticide residue levels. Such characteristics may involve traces of antibiotics, which are typically not prevalent in organic animal products due to stricter regulations on antibiotic use in organic livestock farming (7, 27, 28). According to a meta-analysis of five randomized controlled trials and seven observational studies, antibiotic use is a significant risk factor for gut microbiota dysbiosis in children (29). Furthermore, previous observational studies have indicated that prenatal antibiotic exposure has a stronger association with CD than with UC development (30, 31), which also corresponds with our findings regarding maternal organic food consumption and its link to the offspring’s risk of CD but not UC.

Another explanation for our findings may be the potential nutritional differences between organic and conventional foods, especially considering that maternal nutrition is essential to fetal immune development and gut microbiota maturation (32, 33). These differences may include higher levels of omega-3 polyunsaturated fatty acids in organic dairy products compared to conventional dairy products, as reported by a meta-analysis of 29 studies conducted across multiple countries (34). Similarly, biochemical analyses have shown that organic eggs also tend to have higher omega-3 polyunsaturated fatty acid contents than conventionally produced eggs (35). This might be noteworthy given the immunomodulatory properties of omega-3 polyunsaturated fatty acids (36), which are transferred to the fetus through the umbilical cord blood (37), and thereby, may affect susceptibility to immune-mediated diseases (38, 39). Nonetheless, omega-3 polyunsaturated fatty acids represent just one of several differences between organic and conventional foods. Other examples are improved protein profiles or higher concentrations of vitamin E, iron, and antioxidants (40, 41), which, collectively, might have contributed to the observed association.

Supporting our findings, previous observational studies also found potential health benefits of organic food consumption. In adults, frequent consumption has been linked to lower risks of chronic diseases like obesity, type 2 diabetes, and hypertension (42). Additionally, organic food consumption during pregnancy may lower maternal risks of pre-eclampsia and gestational diabetes, as well as offspring’s risks of hypospadias (43, 44) and otitis media (45). However, evidence in the area remains limited and inconclusive, partly because of the possibility of confounding, which may arise because organic food consumers typically deviate from the general population in terms of their socio-economic status and overall lifestyle (13). This tendency was also observed in our study, where mothers with more frequent organic food consumption generally were more educated and exhibited healthier behaviors, including lower rates of alcohol intake and smoking, as well as higher diet quality. As a result, it is challenging to disentangle the specific effects of organic foods from the broader dietary pattern and lifestyle that they are often associated with Petersen et al., Baudry et al., and Wos et al. (13, 46, 47). After adjusting for these potential confounders, including maternal diet quality, offspring’s CD risk remained significantly lower, which further supports a protective effect of maternal organic food consumption during pregnancy. Yet, it cannot be excluded that some residual confounding might be present from other lifestyle factors, such as physical activity, stress, and unmeasured aspects of maternal diet.

We found no link between maternal diet quality during pregnancy, quantified with the Healthy Eating Index, and offspring’s risk of IBD, including both CD and UC. This indicates that if the observed association between organic food consumption and offspring’s CD risk was confounded by a healthier maternal dietary pattern, it would involve aspects beyond intakes of sugar, salt, saturated fat, dietary fiber, and other components captured by the Healthy Eating Index. Another explanation is that the maternal diet quality was insufficient to exert a measurable influence on offspring’s IBD development, as indicated by the median score of approximately 22, which is considerably lower than the maximum of 80. This finding was unexpected, as pregnant mothers typically are more aware of their dietary habits (48). However, the low diet quality scores likely result from the strict scoring criteria used to quantify the Healthy Eating Index, where points were only awarded for full compliance with the Danish official dietary guidelines (15). The index was based on Danish dietary guidelines from 2017, which differ slightly from those that were in place during the data collection period. In 1994–2005, the applicable dietary guidelines included (1) eating plenty of bread and cereals, (2) eating fruit and vegetables daily, (3) eating rice, pasta, or potatoes daily, (4) eating fish and fish products often, (5) choosing low-fat dairy products and cheese, (6) choosing lean meats and cold cuts, (7) using only small amounts of butter, margarine, and oil, and limiting intakes of salt and sugar (49). Although these guidelines have been updated since, many of their core principles have remained consistent over time. Therefore, the temporal mismatch is unlikely to explain the low diet quality among the mothers and its lack of association with offspring’s disease risk. In addition, using more recent guidelines to construct the index provides a more valid and current measure of diet quality, whereas relying on outdated dietary recommendations could obscure meaningful associations. Moreover, a similar birth cohort study from Norway also reported no association between maternal diet quality and offspring’s CD and UC risk, despite their higher median diet quality score of 81.7 out of 130 (6). This consistency across the two cohorts suggests that maternal diet quality during pregnancy is not a key determinant of IBD risk in offspring, but further research is needed to establish the exact reason for this, as the complex relationship between maternal diet and offspring’s IBD development remains poorly understood.

Our study has some key strengths. To our knowledge, no study has previously investigated the role of organic food consumption in IBD development, which highlights the importance of our findings, especially given the rapidly growing market for organic foods with Denmark and other Nordic countries at the forefront (50). The analyses allowed us to capture both the broader effects of overall organic food intake as well as the differential effects of individual organic food types. This enhanced the specificity and interpretability of our findings through a more nuanced understanding of maternal organic food consumption and its impact on offspring’s IBD development. The prospective assessment of maternal diet during mid-pregnancy minimized the possibility of reverse causality since information on maternal diet was obtained before the offspring developed IBD. Another strength of the study was its population-based design with inclusion of more than 60,000 mother–child pairs from all over Denmark, which makes our findings applicable to the general population. Furthermore, the Danish patient registries contain information on all medical examinations and treatments during the last 40 years. Using register-based data to identify offspring with an IBD diagnosis ensured that all mother–child pairs were followed from childbirth to the offspring’s 18th birthday, and thereby, limited attrition bias due to follow-up losses.

The study also has some potential limitations. The possibility of residual confounding cannot be excluded due to the observational study design, despite collecting detailed information on several confounders, which were thoroughly adjusted for in the analyses. Another limitation of the study is the low number of children with IBD, which may contribute to the large effect sizes. Also, maternal organic food consumption was assessed with self-reported questionnaires using response options that might be interpreted subjectively, which could lead to some degree of subjective variability. However, because the questionnaire data was collected before the offspring developed IBD, any misreporting is likely non-differential concerning the outcome and would attenuate associations toward the null rather than skew them in either direction. Finally, foods in Denmark, including the conventional options, generally contain low levels of pesticide residues (51). This may help explain the absent associations regarding maternal intakes of organic fruit, vegetables, and cereals, as their pesticide residue levels may not deviate enough from conventional options to markedly affect IBD development in offspring. Therefore, different results might be shown in countries with other regulations on permitted pesticide residue levels in food production, but this needs further investigation to be confirmed. Unfortunately, we did not have information on whether the consumed foods were locally produced or imported. Although Denmark follows common regulations for organic food production as a member of the European Union, differences in farming practices and country of origin may still cause variability in the nutritional composition and pesticide residue levels of organic foods (40). This, in turn, could have introduced some degree of variation in exposure levels among organic food consumers, potentially influencing our findings.

In conclusion, this nationwide prospective cohort study of more than 60,000 mother–child pairs from Denmark found no association between maternal organic food consumption during pregnancy and offspring’s risk of pediatric-onset IBD. However, a 60% lower risk of CD was observed among offspring whose mothers had frequent organic food consumption during pregnancy, particularly organic eggs and dairy, whereas no association was found with the risk of UC. Furthermore, maternal consumption of organic meat, fruit, vegetables, and cereals was not associated with disease risk in offspring. Likewise, maternal diet quality was also unrelated to the offspring’s disease risk, suggesting that the protective effect of organic eggs and dairy was unlikely to be explained by confounding from a higher diet quality among the frequent organic food consumers. To our knowledge, this study was the first to examine the relationship between maternal organic food consumption and IBD in offspring, which highlights the importance of its findings given the rapidly growing market for organic foods worldwide. Nevertheless, further research is needed to elucidate the mechanisms underlying these observed associations, as knowledge about the complex relationship between maternal diet during pregnancy and IBD development in offspring remains scarce.

## Data Availability

The data analyzed in this study is subject to the following licenses/restrictions: the data for this study cannot be shared publicly because of the privacy protection of participants within the Danish National Birth Cohort (DNBC). Researchers who wish to use DNBC data are asked to apply for access through the DNBC Secretariat (https://www.dnbc.dk). The study protocol and statistical analysis plan can be made available upon request. Requests to access these datasets should be directed to https://www.dnbc.dk.

## References

[1] Wang R Li Z Liu S Zhang D. Global, regional and national burden of inflammatory bowel disease in 204 countries and territories from 1990 to 2019: a systematic analysis based on the global burden of disease study 2019. BMJ Open. (2023) 13:e065186. doi: 10.1136/bmjopen-2022-065186, PMID: 36977543 PMC10069527

[2] Wang Y Pan CW Huang Y Zheng X Li S He M. Global epidemiology and geographic variations of pediatric-onset inflammatory bowel disease: a comprehensive analysis of the global burden of disease study 1990 to 2019. Inflamm Bowel Dis. (2025) 31:376–85. doi: 10.1093/ibd/izae093, PMID: 38676392

[3] Piovani D Danese S Peyrin-Biroulet L Nikolopoulos GK Lytras T Bonovas S. Environmental risk factors for inflammatory bowel diseases: an umbrella review of Meta-analyses. Gastroenterology. (2019) 157:647–659.e4. doi: 10.1053/j.gastro.2019.04.016, PMID: 31014995

[4] Estevinho MM Midya V Cohen-Mekelburg S Allin KH Fumery M Pinho SS. Emerging role of environmental pollutants in inflammatory bowel disease risk, outcomes and underlying mechanisms. Gut. (2025) 74:477–86. doi: 10.1136/gutjnl-2024-332523, PMID: 39179372 PMC11802320

[5] Huang C Tan H Song M Liu K Liu H Wang J. Maternal Western diet mediates susceptibility of offspring to Crohn's-like colitis by deoxycholate generation. Microbiome. (2023) 11:96. doi: 10.1186/s40168-023-01546-6, PMID: 37131223 PMC10155335

[6] Guo A Brantsæter AL Borge TC M E Imberg H Mårild K. Maternal diet in pregnancy and the risk of inflammatory bowel disease in the offspring: a prospective cohort study. Am J Clin Nutr. (2025) 121:32–9. doi: 10.1016/j.ajcnut.2024.10.017, PMID: 39461723 PMC11747187

[7] The European Parliament and the Council of The European Union. Regulation (EU) 2018/848 of the European Parliament and of the council of 30 may 2018 on organic production and labelling of organic products and repealing council regulation (EC) no 834/2007. Off J Eur Union. (2018) 61, 1–92.

[8] Yan S Tian S Meng Z Teng M Sun W Jia M. Exposure to nitenpyram during pregnancy causes colonic mucosal damage and non-alcoholic steatohepatitis in mouse offspring: the role of gut microbiota. Environ Pollut. (2021) 271:116306. doi: 10.1016/j.envpol.2020.116306, PMID: 33360580

[9] Djekkoun N Depeint F Guibourdenche M Sabbouri HEKE Corona A Rhazi L. Perigestational exposure of a combination of a high-fat diet and pesticide impacts the metabolic and microbiotic status of dams and pups; a preventive strategy based on prebiotics. Eur J Nutr. (2023) 62:1253–65. doi: 10.1007/s00394-022-03063-y, PMID: 36510012

[10] Jin G Tang Q Ma J Liu X Zhou B Sun Y. Maternal emulsifier P80 intake induces gut dysbiosis in offspring and increases their susceptibility to colitis in adulthood. mSystems. (2021) 6:e01337–20. doi: 10.1128/mSystems.01337-20, PMID: 33727402 PMC8547008

[11] Agrawal M Sabino J Frias-Gomes C Hillenbrand CM Soudant C Axelrad JE. Early life exposures and the risk of inflammatory bowel disease: systematic review and meta-analyses. EClinicalMedicine. (2021) 36:884. doi: 10.1016/j.eclinm.2021.100884, PMID: 34308303 PMC8257976

[12] Soroka A Wojciechowska-Solis J. Consumer motivation to buy organic food depends on lifestyle. Foods. (2019) 8:581. doi: 10.3390/foods8110581, PMID: 31744098 PMC6915680

[13] Petersen SB Rasmussen MA Strøm M Halldorsson TI Olsen SF. Sociodemographic characteristics and food habits of organic consumers--a study from the Danish National Birth Cohort. Public Health Nutr. (2013) 16:1810–9. doi: 10.1017/S1368980012004119, PMID: 22971358 PMC10271409

[14] Bjerregaard AA Halldorsson TI Tetens I Olsen SF. Mother's dietary quality during pregnancy and offspring's dietary quality in adolescence: follow-up from a national birth cohort study of 19,582 mother-offspring pairs. PLoS Med. (2019) 16:e1002911. doi: 10.1371/journal.pmed.1002911, PMID: 31513597 PMC6742222

[15] Ministry of Food, Agriculture and Fisheries of Denmark The official dietary guidelines Glostrup, Denmark The Danish Veterinary and Food Administration (2021). Available online at: https://foedevarestyrelsen.dk/Media/638198073401130807/Danish_Official_Dietary_Guidelines_Good_for_Health_and_climate_2021_SCRE.pdf (Accessed August 19, 2024).

[16] Sundhedsstyrelsen. Sunde vaner før, under og efter graviditet. Denmark: Sundhedsstyrelsen og Komiteen for Sundhedsoplysning, (2022). Available online at: https://www.sst.dk/-/media/Udgivelser/2022/Foerste-1000-dage/Sunde_vaner_8_-udgave-2022.ashx?sc_lang=da&hash=49B4219C43E3BFB5C2CDB4273D3F2668 (Accessed August 19, 2024).

[17] Olsen SF Mikkelsen TB Knudsen VK Orozova-Bekkevold I Halldórsson TI Strøm M. Data collected on maternal dietary exposures in the Danish National Birth Cohort. Paediatr Perinat Epidemiol. (2007) 21:76–86. doi: 10.1111/j.1365-3016.2007.00777.x, PMID: 17239183

[18] Agrawal M Christensen HS Bøgsted M Colombel JF Jess T Allin KH. The rising burden of inflammatory bowel disease in Denmark over two decades: a Nationwide cohort study. Gastroenterology. (2022) 163:1547–1554.e5. doi: 10.1053/j.gastro.2022.07.062, PMID: 35952799 PMC9691534

[19] Tetens I Pedersen AN Schwab U Fogelholm M Þórsdóttir I Gunnarsdóttir I. Nordic nutrition recommendations 2012 Copenhagen, Denmark: integrating nutrition and physical activity Nordic Council of Ministers (2014).

[20] UNESCO Institute for Statistics. International standard classification of education. (2012) Montreal, Canada: UNESCO Institute for Statistics, Available online at: https://uis.unesco.org/sites/default/files/documents/international-standard-classification-of-education-isced-2011-en.pdf (Accessed October 30, 2024).

[21] Qiu P Ishimoto T Fu L Zhang J Zhang Z Liu Y. The gut microbiota in inflammatory bowel disease. Front Cell Infect Microbiol. (2022) 12:733992. doi: 10.3389/fcimb.2022.733992, PMID: 35273921 PMC8902753

[22] Gois MFB Fernández-Pato A Huss A Gacesa R Wijmenga C Weersma RK. Impact of occupational pesticide exposure on the human gut microbiome. Front Microbiol. (2023) 14:1223120. doi: 10.3389/fmicb.2023.1223120, PMID: 37637104 PMC10448898

[23] Gómez-Ramos MDM Nannou C Martínez Bueno MJ Goday A Murcia-Morales M Ferrer C. Pesticide residues evaluation of organic crops. A critical appraisal. Food Chem X. (2020) 5:100079. doi: 10.1016/j.fochx.2020.100079, PMID: 32083251 PMC7019120

[24] Lu X Shi Z Jiang L Zhang S. Maternal gut microbiota in the health of mothers and offspring: from the perspective of immunology. Front Immunol. (2024) 15:1362784. doi: 10.3389/fimmu.2024.1362784, PMID: 38545107 PMC10965710

[25] Murphy KM Le SM Wilson AE Warner DA. The microbiome as a maternal effect: a systematic review on vertical transmission of microbiota. Integr Comp Biol. (2023) 63:597–609. doi: 10.1093/icb/icad031, PMID: 37218690

[26] Mirpuri J. Evidence for maternal diet-mediated effects on the offspring microbiome and immunity: implications for public health initiatives. Pediatr Res. (2021) 89:301–6. doi: 10.1038/s41390-020-01121-x, PMID: 32919391 PMC7897208

[27] Zakaria AM Elmeligy E Abdulkarim A Mohammed HH Khalphallah A Ali MA. Monitoring antimicrobial residues in table eggs in Aswan governorate markets and their impact on egg quality and public health. Open Vet J. (2023) 13:523–31. doi: 10.5455/OVJ.2023.v13.i5.4, PMID: 37304600 PMC10257451

[28] Welsh JA Braun H Brown N Um C Ehret K Figueroa J. Production-related contaminants (pesticides, antibiotics and hormones) in organic and conventionally produced milk samples sold in the USA. Public Health Nutr. (2019) 22:2972–80. doi: 10.1017/S136898001900106X, PMID: 31238996 PMC6792142

[29] McDonnell L Gilkes A Ashworth M Rowland V Harries TH Armstrong D. Association between antibiotics and gut microbiome dysbiosis in children: systematic review and meta-analysis. Gut Microbes. (2021) 13:1–18. doi: 10.1080/19490976.2020.1870402, PMID: 33651651 PMC7928022

[30] Örtqvist AK Lundholm C Halfvarson J Ludvigsson JF Almqvist C. Fetal and early life antibiotics exposure and very early onset inflammatory bowel disease: a population-based study. Gut. (2019) 68:218–25. doi: 10.1136/gutjnl-2017-314352, PMID: 29321166

[31] Canova C Ludvigsson JF Di Domenicantonio R Zanier L Barbiellini Amidei C Zingone F. Perinatal and antibiotic exposures and the risk of developing childhood-onset inflammatory bowel disease: a nested case-control study based on a population-based birth cohort. Int J Environ Res Public Health. (2020) 17:2409. doi: 10.3390/ijerph17072409, PMID: 32252276 PMC7177699

[32] Nelson BN Friedman JE. Developmental programming of the fetal immune system by maternal Western-style diet: mechanisms and implications for disease pathways in the offspring. Int J Mol Sci. (2024) 25:5951. doi: 10.3390/ijms25115951, PMID: 38892139 PMC11172957

[33] Maher SE O’Brien EC Moore RL Byrne DF Geraghty AA Saldova R. The association between the maternal diet and the maternal and infant gut microbiome: a systematic review. Br J Nutr. (2023) 129:1491–9. doi: 10.1017/S0007114520000847 32129734

[34] Palupi E Jayanegara A Ploeger A Kahl J. Comparison of nutritional quality between conventional and organic dairy products: a meta-analysis. J Sci Food Agric. (2012) 92:2774–81. doi: 10.1002/jsfa.5639, PMID: 22430502

[35] Mesas AE Fernández-Rodríguez R Martínez-Vizcaíno V López-Gil JF Fernández-Franco S Bizzozero-Peroni B. Organic egg consumption: a systematic review of aspects related to human health. Front Nutr. (2022) 9:937959. doi: 10.3389/fnut.2022.937959, PMID: 35811992 PMC9263557

[36] Bodur M Yilmaz B Agagunduz D Ozogul Y. Immunomodulatory effects of Omega-3 fatty acids: mechanistic insights and health implications. Mol Nutr Food Res. (2025) 69:e202400752. doi: 10.1002/mnfr.202400752, PMID: 40159804 PMC12087734

[37] Miles EA Childs CE Calder PC. Long-chain polyunsaturated fatty acids (LCPUFAs) and the developing immune system: a narrative review. Nutrients. (2021) 13:247. doi: 10.3390/nu13010247, PMID: 33467123 PMC7830895

[38] Best KP Gold M Kennedy D Martin J Makrides M. Omega-3 long-chain PUFA intake during pregnancy and allergic disease outcomes in the offspring: a systematic review and meta-analysis of observational studies and randomized controlled trials. Am J Clin Nutr. (2016) 103:128–43. doi: 10.3945/ajcn.115.111104, PMID: 26675770

[39] Huynh LBP Nguyen NN Fan HY Huang SY Huang CH Chen YC. Maternal Omega-3 supplementation during pregnancy, but not childhood supplementation, reduces the risk of food allergy diseases in offspring. J Allergy Clin Immunol Pract. (2023) 11:2862–2871.e8. doi: 10.1016/j.jaip.2023.06.005, PMID: 37301431

[40] Prache S Lebret B Baéza E Martin B Gautron J Feidt C. Review: quality and authentication of organic animal products in Europe. Animal. (2022) 16:100405. doi: 10.1016/j.animal.2021.100405 34844891

[41] Popa ME Mitelut AC Popa EE Stan A Popa VI. Organic foods contribution to nutritional quality and value. Trends Food Sci Technol. (2019) 84:15–8. doi: 10.1016/j.tifs.2018.01.003

[42] Poulia KA Bakaloudi DR Alevizou M Papakonstantinou E Zampelas A Chourdakis M. Impact of organic foods on chronic diseases and health perception: a systematic review of the evidence. Eur J Clin Nutr. (2025) 79:90–103. doi: 10.1038/s41430-024-01505-w, PMID: 39261657

[43] Christensen JS Asklund C Skakkebæk NE Jørgensen N Andersen HR Jørgensen TM. Association between organic dietary choice during pregnancy and hypospadias in offspring: a study of mothers of 306 boys operated on for hypospadias. J Urol. (2013) 189:1077–82. doi: 10.1016/j.juro.2012.09.116, PMID: 23036983

[44] Brantsæter AL Torjusen H Meltzer HM Papadopoulou E Hoppin JA Alexander J. Organic food consumption during pregnancy and hypospadias and cryptorchidism at birth: the Norwegian mother and child cohort study (MoBa). Environ Health Perspect. (2016) 124:357–64. doi: 10.1289/ehp.1409518, PMID: 26307850 PMC4786987

[45] Buscail C Chevrier C Serrano T Pelé F Monfort C Cordier S. Prenatal pesticide exposure and otitis media during early childhood in the PELAGIE mother-child cohort. Occup Environ Med. (2015) 72:837–44. doi: 10.1136/oemed-2015-103039, PMID: 26347056

[46] Baudry J Allès B Péneau S Touvier M Méjean C Hercberg S. Dietary intakes and diet quality according to levels of organic food consumption by French adults: cross-sectional findings from the NutriNet-santé cohort study. Public Health Nutr. (2017) 20:638–48. doi: 10.1017/S1368980016002718, PMID: 27731291 PMC10261570

[47] Woś K Dobrowolski H Gajewska D Rembiałkowska E. Diet quality indicators and organic food consumption in mothers of young children. J Sci Food Agric. (2025) 105:3900–10. doi: 10.1002/jsfa.14143, PMID: 39868842

[48] Olloqui-Mundet MJ Cavia MDM Alonso-Torre SR Carrillo C. Dietary habits and nutritional knowledge of pregnant women: the importance of nutrition education. Foods. (2024) 13:3189. doi: 10.3390/foods13193189, PMID: 39410224 PMC11475029

[49] Astrup A Andersen NL Stender S Trolle E. Kostrådene (2025). Available at https://ugeskriftet.dk/videnskab/kostradene-2005 (Accessed June 12, 2025). 15962862

[50] Pekala A. Market analysis of organic foods in the Nordic and Baltic countries. TemaNord. (2019) 540:1–138. doi: 10.6027/TN2019-540

[51] Poulsen ME Andersen JH Petersen A Hartkopp H. Pesticides: part 2. Danish veterinary and food administration In. Food monitoring, 1998–2003. Copenhagen: DTU Fødevareinstituttet Danish Veterinary and Food Administration. Søborg, Denmark. (2005)

