# Supplementary material for: Impact of maternal organic food consumption and diet quality during pregnancy on offspring’s risk of inflammatory bowel disease: findings from a Danish National Birth Cohort Study
Source: Front Nutr. 2025 Jul 10;12:1632729. doi: 10.3389/fnut.2025.1632729 (PMC12286796; doi:10.3389/fnut.2025.1632729)
Supplement: Supplementary file 1 [file Data_Sheet_1.docx]

Supplementary Material

[**Supplementary Figure 1.** Kaplan-Meier curves of the cumulative incidence for pediatric-onset (≤18 years old) inflammatory bowel disease in offspring in the Danish National Birth Cohort. 2](#_Toc200634426)

[**Supplementary Figure 2.** Histogram with the distribution of Healthy Eating Index scores. X-axis: Healthy Eating Index score (ranging from 0 to 80); Y-axis: Proportion of mothers with the score (count); The dashed line illustrates the median score. 3](#_Toc200634427)

[**Supplementary Figure 3.** Histogram with the distribution of scores for components comprising the Healthy Eating Index. X-axis: Scores for individual components comprising the Healthy Eating Index (ranging from 0 to 10); Y-axis: Proportion of mothers with the score (count). 4](#_Toc200634428)

[**Supplementary Table 1.** Scoring criteria used for the Healthy Eating Index.^1^ 5](#_Toc200634429)

[S**upplementary Table 2.** Baseline characteristics in the excluded compared to the included mother-child pairs. 6](#_Toc200634430)

[S**upplementary Table 3. A**ssociations of maternal organic food consumption^1^ during pregnancy with the risk of pediatric-onset inflammatory bowel disease in offspring, accounting for siblings in the cohort by only including the firstborn sibling in case the mother participated in the DNBC with more than one pregnancy. 8](#_Toc200634431)

[**Supplementary Table 4.** Associations of maternal organic egg consumption^1^ during pregnancy with the risk of pediatric-onset inflammatory bowel disease in offspring. 9](#_Toc200634432)

[**Supplementary Table 5.** Associations of maternal organic dairy consumption^1^ during pregnancy with the risk of pediatric-onset inflammatory bowel disease in offspring. 10](#_Toc200634433)

[**Supplementary Table 6.** Associations of maternal organic meat consumption^1^ during pregnancy with the risk of pediatric-onset inflammatory bowel disease in offspring. 11](#_Toc200634434)

[**Supplementary Table 7.** Associations of maternal organic fruit consumption^1^ during pregnancy with the risk of pediatric-onset inflammatory bowel disease in offspring. 12](#_Toc200634435)

[**Supplementary Table 8.** Associations of maternal organic vegetable consumption^1^ during pregnancy with the risk of pediatric-onset inflammatory bowel disease in offspring. 13](#_Toc200634436)

[**Supplementary Table 9.** Associations of maternal organic cereal consumption^1^ during pregnancy with the risk of pediatric-onset inflammatory bowel disease in offspring. 14](#_Toc200634437)


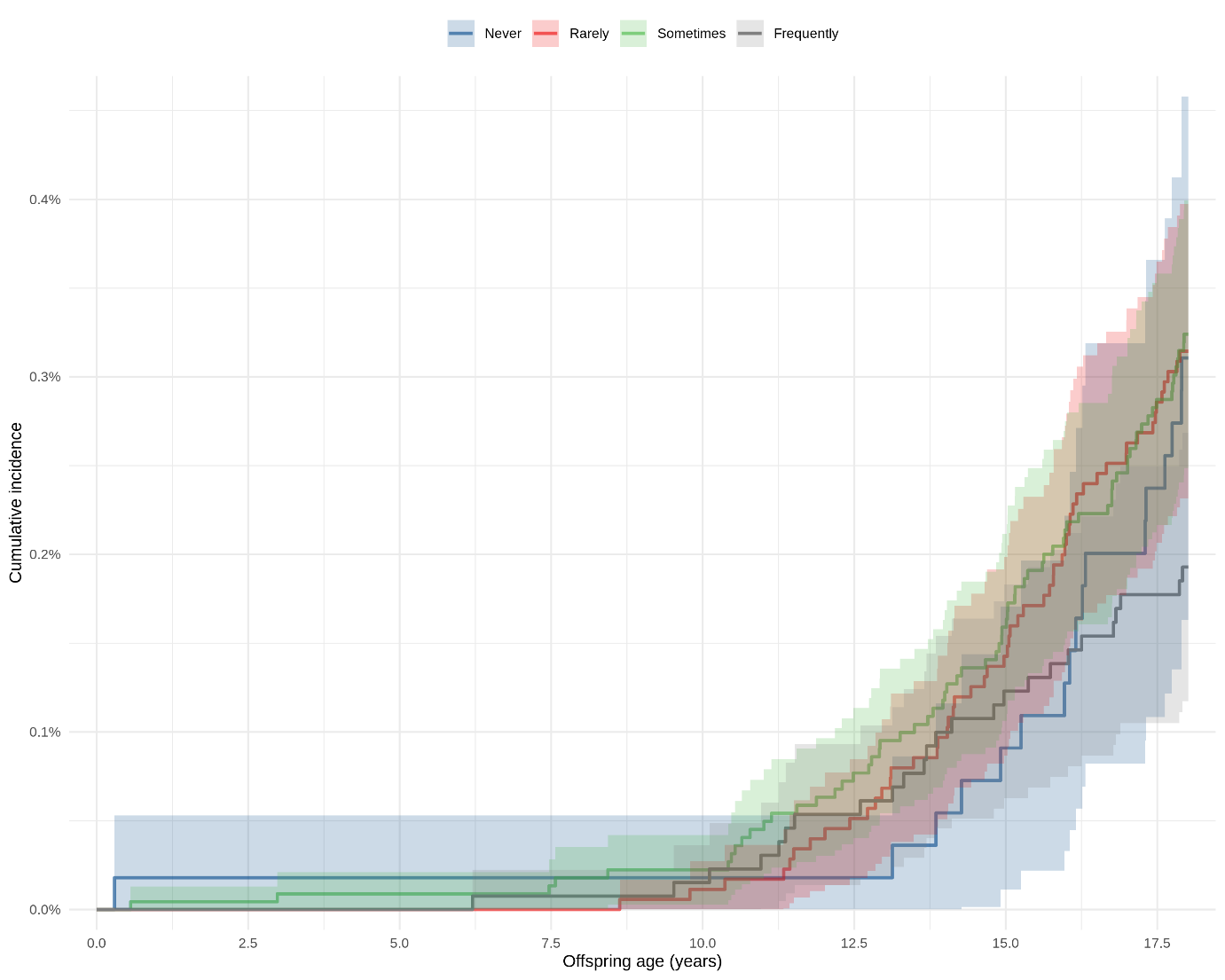


**Supplementary Figure 1.** Kaplan-Meier curves of the cumulative incidence for pediatric-onset (≤18 years old) inflammatory bowel disease in offspring in the Danish National Birth Cohort.


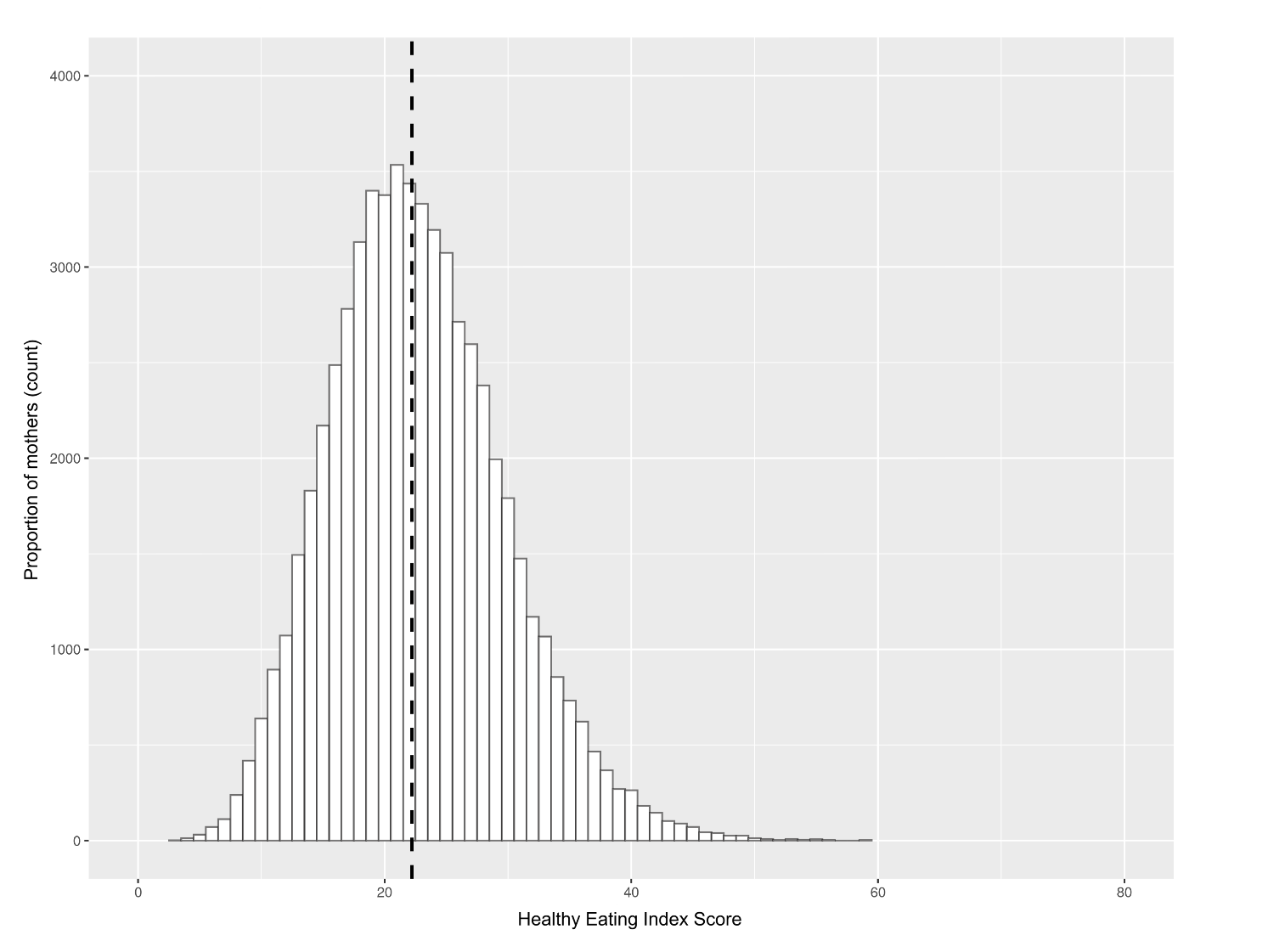


**Supplementary Figure 2.** Histogram with the distribution of Healthy Eating Index scores. X-axis: Healthy Eating Index score (ranging from 0 to 80); Y-axis: Proportion of mothers with the score (count); The dashed line illustrates the median score.


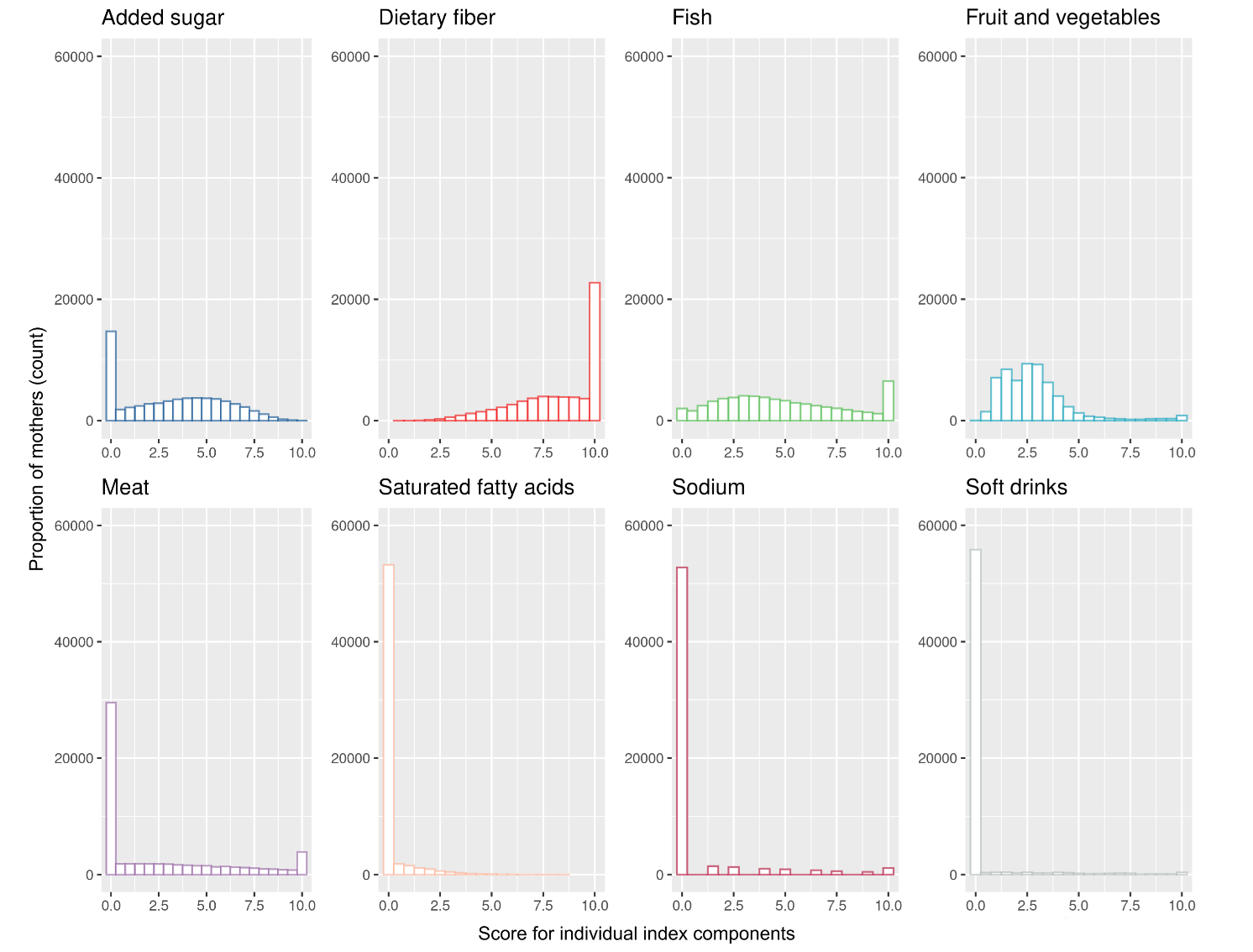


**Supplementary Figure 3.** Histogram with the distribution of scores for components comprising the Healthy Eating Index. X-axis: Scores for individual components comprising the Healthy Eating Index (ranging from 0 to 10); Y-axis: Proportion of mothers with the score (count).

# Supplementary Table 1. Scoring criteria used for the Healthy Eating Index.^1^

| **Component** | **Scores** | **Recommendation** |
| --- | --- | --- |
| Fruits and vegetables | 0 g = 0 points ≥600 g = 10 points | ≥600 g/day, where at minimum half is vegetables |
| Dietary fibers | 0 g = 0 points ≥30 g = 10 points | ≥30 g/day |
| Fish | 0 g = 0 points ≥350 g = 10 points | ≥350 g/week |
| Red meat | >500 g = 0 points ≤200 g = 10 points | ≤500 g/week |
| Saturated fatty acids | ≥10E% = 0 points 0E% = 10 points | <10 E% |
| Sodium | >2.4 g = 0 points ≤1.6 g = 10 points | ≤2.4 g/day |
| Sugar-sweetened beverages | >500 g = 0 points 0 g = 10 points | ≤500 ml/week |
| Added sugar | ≥10E% = 0 points 0E% = 10 points | <10 E% |

^1^The table is modified from Bjerregaard et al (1). The scoring is continuous for intakes between the minimum and maximum cutoff for intakes. Recommendations for intakes of fruits, vegetables, dietary fibers, fish, red meat, and sugar-sweetened beverages are from Ministry of Food, Agriculture and Fisheries of Denmark (2), and recommendations for intakes of saturated fatty acids, sodium, and added sugar are from Nordic Nutrition Recommendation (3).

# Supplementary Table 2. Baseline characteristics in the excluded compared to the included mother-child pairs.

|  | **Total**  **(n=96817)** | **Excluded**  **(n=36570)** | **Included**  **(n=60247)** | **p-value^1^** |
| --- | --- | --- | --- | --- |
| Organic food consumption |  |  |  | <0.001 |
| Never, n (%) | 9317 (9.6%) | 3714 (10.2%) | 5603 (9.3%) |  |
| Rarely, n (%) | 26251 (27.1%) | 8190 (22.4%) | 18061 (30.0%) |  |
| Sometimes, n (%) | 33240 (34.3%) | 10399 (28.4%) | 22841 (37.9%) |  |
| Frequently, n (%) | 19969 (20.6%) | 6227 (17.0%) | 13742 (22.8%) |  |
| Missing, n (%) | 8040 (8.3%) | 8040 (22.0%) | 0 (0.0%) |  |
| Organic egg consumption^2^ |  |  |  | 0.047 |
| Low, n (%) | 39915 (41.2%) | 7082 (19.4%) | 32833 (54.5%) |  |
| High, n (%) | 33416 (34.5%) | 6002 (16.4%) | 27414 (45.5%) |  |
| Missing, n (%) | 23486 (24.3%) | 23486 (64.2%) | 0 (0.0%) |  |
| Organic dairy consumption^2^ |  |  |  | 0.041 |
| Low, n (%) | 43682 (45.1%) | 7774 (21.3%) | 35908 (59.6%) |  |
| High, n (%) | 29770 (30.7%) | 5431 (14.9%) | 24339 (40.4%) |  |
| Missing, n (%) | 23365 (24.1%) | 23365 (63.9%) | 0 (0.0%) |  |
| Organic meat consumption^2^ |  |  |  | <0.001 |
| Low, n (%) | 66359 (68.5%) | 11532 (31.5%) | 54827 (91.0%) |  |
| High, n (%) | 6741 (7.0%) | 1321 (3.6%) | 5420 (9.0%) |  |
| Missing, n (%) | 23717 (24.5%) | 23717 (64.9%) | 0 (0.0%) |  |
| Organic fruit consumption^2^ |  |  |  | <0.001 |
| Low, n (%) | 63004 (65.1%) | 11018 (30.1%) | 51986 (86.3%) |  |
| High, n (%) | 10273 (10.6%) | 2012 (5.5%) | 8261 (13.7%) |  |
| Missing, n (%) | 23540 (24.3%) | 23540 (64.4%) | 0 (0.0%) |  |
| Organic vegetable consumption^2^ |  |  |  | <0.001 |
| Low, n (%) | 55511 (57.3%) | 9681 (26.5%) | 45830 (76.1%) |  |
| High, n (%) | 17754 (18.3%) | 3337 (9.1%) | 14417 (23.9%) |  |
| Missing, n (%) | 23552 (24.3%) | 23552 (64.4%) | 0 (0.0%) |  |
| Organic cereal consumption^2^ |  |  |  | <0.001 |
| Low, n (%) | 54058 (55.8%) | 9416 (25.7%) | 44642 (74.1%) |  |
| High, n (%) | 19149 (19.8%) | 3544 (9.7%) | 15605 (25.9%) |  |
| Missing, n (%) | 23610 (24.4%) | 23610 (64.6%) | 0 (0.0%) |  |
| Maternal diet quality |  |  |  | <0.001 |
| Median (IQR) | 22.3 (17.9-27.3) | 22.8 (18.0-27.8) | 22.2 (17.8-27.2) |  |
| Missing, n (%) | 23011 (23.8%) | 23011 (62.9%) | 0 (0.0%) |  |
| Maternal age at birth (years) |  |  |  | <0.001 |
| Median (IQR) | 30.0 (27.0-33.0) | 30.0 (27.0-33.0) | 30.0 (27.0-33.0) |  |
| Missing, n (%) | 0 (0·0%) | 0 (0·0%) | 0 (0·0%) |  |
| Maternal educational level |  |  |  | <0.001 |
| Lower education, n (%) | 11674 (12.1%) | 4349 (11.9%) | 7325 (12.2%) |  |
| Medium education, n (%) | 35805 (37.0%) | 12151 (33.2%) | 23654 (39.3%) |  |
| Higher education, n (%) | 42099 (43.5%) | 12831 (35.1%) | 29268 (48.6%) |  |
| Missing, n (%) | 7239 (7.5%) | 7239 (19.8%) | 0 (0.0%) |  |
| Maternal pre-pregnancy body mass index (kg/m^2^) |  |  |  | <0.001 |
| Median (IQR) | 22.6 (20.7-25.4) | 22.7 (20.7-25.6) | 22.6 (20.7-25.4) |  |
| Missing, n (%) | 7591 (7.8%) | 7591 (20.8%) | 0 (0.0%) |  |
| Maternal smoking |  |  |  | <0.001 |
| No, n (%) | 71858 (74.2%) | 21343 (58.4%) | 50515 (83.8%) |  |
| Yes, n (%) | 15933 (16.5%) | 6201 (17.0%) | 9732 (16.2%) |  |
| Missing, n (%) | 9026 (9.3%) | 9026 (24.7%) | 0 (0.0%) |  |
| Maternal nutritional supplement use |  |  |  | <0.001 |
| No, n (%) | 3026 (3.1%) | 1750 (4.8%) | 1276 (2.1%) |  |
| Yes, n (%) | 85751 (88.6%) | 26780 (73.2%) | 58971 (97.9%) |  |
| Missing, n (%) | 8040 (8.3%) | 8040 (22.0%) | 0 (0.0%) |  |
| Maternal alcohol intake |  |  |  | <0.001 |
| No, n (%) | 44538 (46.0%) | 14748 (40.3%) | 29790 (49.4%) |  |
| Yes, n (%) | 43276 (44.7%) | 12819 (35.1%) | 30457 (50.6%) |  |
| Missing, n (%) | 9003 (9.3%) | 9003 (24.6%) | 0 (0.0%) |  |
| Maternal antibiotics use |  |  |  | <0.001 |
| 0 courses, n (%) | 66861 (69.1%) | 24622 (67.3%) | 42239 (70.1%) |  |
| 1-2 courses, n (%) | 22819 (23.6%) | 8756 (23.9%) | 14063 (23.3%) |  |
| ≥3 courses, n (%) | 7137 (7.4%) | 3192 (8.7%) | 3945 (6.5%) |  |
| Missing, n (%) | 0 (0.0%) | 0 (0.0%) | 0 (0.0%) |  |
| Parental IBD |  |  |  | 0.117 |
| No, n (%) | 95736 (98.9%) | 36187 (99.0%) | 59549 (98.8%) |  |
| Yes, n (%) | 1081 (1.1%) | 383 (1.0%) | 698 (1.2%) |  |
| Missing, n (%) | 0 (0.0%) | 0 (0.0%) | 0 (0.0%) |  |
| Maternal energy intake (MJ/day) |  |  |  | 0.511 |
| Median (IQR) | 9.8 (8.2-11.6) | 9.8 (8.1-11.7) | 9.8 (8.3-11.6) |  |
| Missing, n (%) | 25462 (26.3%) | 23131 (63.3%) | 2331 (3.9%) |  |
| Offspring sex |  |  |  | 0.524 |
| Girl, n (%) | 47064 (48.6%) | 17772 (48.6%) | 29292 (48.6%) |  |
| Boy, n (%) | 49576 (51.2%) | 18621 (50.9%) | 30955 (51.4%) |  |
| Missing, n (%) | 177 (0.2%) | 177 (0.5%) | 0 (0.0%) |  |
| Offspring's antibiotic use in first year |  |  |  | 0.255 |
| 0 courses, n (%) | 57169 (59.0%) | 21679 (59.3%) | 35490 (58.9%) |  |
| ≥1 courses, n (%) | 39648 (41.0%) | 14891 (40.7%) | 24757 (41.1%) |  |
| Missing, n (%) | 0 (0.0%) | 0 (0.0%) | 0 (0.0%) |  |
| Breastfeeding duration^3^ (days) |  |  |  | <0.001 |
| Median (IQR) | 180 (134-180) | 180 (120-180) | 180 (134-180) |  |
| Missing, n (%) | 26614 (27.5%) | 14877 (40.7%) | 11737 (19.5%) |  |
| Preterm delivery |  |  |  | <0.001 |
| No (≥37 weeks), n (%) | 88945 (91.9%) | 31651 (86.5%) | 57294 (95.1%) |  |
| Yes (<37 weeks), n (%) | 6201 (6.4%) | 4022 (11.0%) | 2179 (3.6%) |  |
| Missing, n (%) | 1671 (1.7%) | 897 (2.5%) | 774 (1.3%) |  |
| Disease subtype |  |  |  | 0.392 |
| No disease, n (%) | 96556 (99.7%) | 36477 (99.7%) | 60079 (99.7%) |  |
| Crohn’s disease, n (%) | 144 (0.2%) | 49 (0.1%) | 95 (0.2%) |  |
| Ulcerative colitis, n (%) | 117 (0.1%) | 44 (0.1%) | 73 (0.1%) |  |

^1^Categorical variables were tested with X^2^ test, and continuous variables were tested with Kruskal-Wallis test. ^2^Intakes of organic food types were modeled as a binary variable (low versus high frequency of consumption) by combining responses ‘never’ and ‘sometimes’ as well as ‘frequently’ and ‘always’, respectively. ^3^Truncated at 180 days. Abbreviations: IBD, inflammatory bowel disease.

Supplementary Table 3. Associations of maternal organic food consumption during pregnancy with the risk of pediatric-onset inflammatory bowel disease in offspring, accounting for siblings in the cohort by only including the firstborn sibling in case the mother participated in the DNBC with more than one pregnancy.

|  | **Never**  **(n=5045)** | **Rarely**  **(n=16270)** | **Sometimes (n=20665)** | **Frequently**  **(n=12455)** |
| --- | --- | --- | --- | --- |
| Inflammatory bowel disease |  |  |  |  |
| Cases, n (%) | 15 (0.30%) | 51 (0.31%) | 64 (0.31%) | 22 (0.18%) |
| Unadjusted HR (95% CI)^1^ | Reference | 1.06 (0.60-1.89) | 1.06 (0.60-1.86) | 0.62 (0.32-1.19) |
| Adjusted HR (95% CI)^2^ | Reference | 1.04 (0.59-1.86) | 1.08 (0.61-1.92) | 0.65 (0.33-1.29) |
| Crohn’s disease |  |  |  |  |
| Cases, n (%) | 10 (0.20%) | 32 (0.20%) | 38 (0.18%) | 9 (0.07%) |
| Unadjusted HR (95% CI)^1^ | Reference | 1.00 (0.49-2.04) | 0.94 (0.47-1.90) | 0.38 (0.15-0.93) |
| Adjusted HR (95% CI)^2^ | Reference | 0.92 (0.45-1.88) | 0.87 (0.43-1.78) | 0.36 (0.14-0.91) |
| Ulcerative colitis |  |  |  |  |
| Cases, n (%) | 5 (0.10%) | 19 (0.11%) | 26 (0.13%) | 13 (0.10%) |
| Unadjusted HR (95% CI)^1^ | Reference | 1.19 (0.44-3.19) | 1.29 (0.50-3.37) | 1.09 (0.39-3.06) |
| Adjusted HR (95% CI)^2^ | Reference | 1.27 (0.47-3.42) | 1.50 (0.57-3.98) | 1.34 (0.46-3.89) |

^1^Cox proportional hazards model; ^2^Cox proportional hazards model adjusted for maternal educational level, diet quality during pregnancy, pre-pregnancy body mass index, smoking during pregnancy, nutritional supplement use during pregnancy, antibiotics use during pregnancy, and parental inflammatory bowel disease diagnosis. Abbreviations: CI, confidence interval; HR, hazard ratio.

Supplementary Table 4. Associations of maternal organic egg consumption^1^ during pregnancy with the risk of pediatric-onset inflammatory bowel disease in offspring.

|  | **Low (n=32833)** | **High (n=27414)** |
| --- | --- | --- |
| Inflammatory bowel disease |  |  |
| Cases, n (%) | 107 (0.33%) | 61 (0.22%) |
| Unadjusted HR (95% CI)^2^ | Reference | 0.70 (0.51-0.95) |
| Adjusted HR (95% CI)^3^ | Reference | 0.70 (0.51-0.97) |
| Crohn’s disease |  |  |
| Cases, n (%) | 64 (0.19%) | 31 (0.11%) |
| Unadjusted HR (95% CI)^2^ | Reference | 0.59 (0.38-0.91) |
| Adjusted HR (95% CI)^3^ | Reference | 0.58 (0.37-0.90) |
| Ulcerative colitis |  |  |
| Cases, n (%) | 43 (0.13%) | 30 (0.11%) |
| Unadjusted HR (95% CI)^2^ | Reference | 0.85 (0.53-1.36) |
| Adjusted HR (95% CI)^3^ | Reference | 0.88 (0.55-1.43) |

^1^Maternal intake of organic eggs was modeled as a binary variable (low versus high frequency of consumption) by combining responses ‘never’ and ‘sometimes’ as well as ‘frequently’ and ‘always’, respectively; ^2^Cox proportional hazards model; ^3^Cox proportional hazards model adjusted for maternal educational level, diet quality during pregnancy, pre-pregnancy body mass index, smoking during pregnancy, nutritional supplement use during pregnancy, antibiotics use during pregnancy, and parental inflammatory bowel disease diagnosis. Abbreviations: CI, confidence interval; HR, hazard ratio.

# Supplementary Table 5. Associations of maternal organic dairy consumption^1^ during pregnancy with the risk of pediatric-onset inflammatory bowel disease in offspring.

|  | **Low (n=35908)** | **High (n=24339)** |
| --- | --- | --- |
| Inflammatory bowel disease |  |  |
| Cases, n (%) | 112 (0.31%) | 56 (0.23%) |
| Unadjusted HR (95% CI)^2^ | Reference | 0.75 (0.55-1.04) |
| Adjusted HR (95% CI)^3^ | Reference | 0.78 (0.56-1.09) |
| Crohn’s disease |  |  |
| Cases, n (%) | 69 (0.19%) | 26 (0.11%) |
| Unadjusted HR (95% CI)^2^ | Reference | 0.57 (0.36-0.89) |
| Adjusted HR (95% CI)^3^ | Reference | 0.56 (0.35-0.90) |
| Ulcerative colitis |  |  |
| Cases, n (%) | 43 (0.12%) | 30 (0.12%) |
| Unadjusted HR (95% CI)^2^ | Reference | 1.05 (0.66-1.68) |
| Adjusted HR (95% CI)^3^ | Reference | 1.15 (0.70-1.88) |

^1^Maternal intake of organic dairy was modeled as a binary variable (low versus high frequency of consumption) by combining responses ‘never’ and ‘sometimes’ as well as ‘frequently’ and ‘always’, respectively; ^2^Cox proportional hazards model; ^3^Cox proportional hazards model adjusted for maternal educational level, diet quality during pregnancy, pre-pregnancy body mass index, smoking during pregnancy, nutritional supplement use during pregnancy, antibiotics use during pregnancy, and parental inflammatory bowel disease diagnosis. Abbreviations: CI, confidence interval; HR, hazard ratio.

# Supplementary Table 6. Associations of maternal organic meat consumption^1^ during pregnancy with the risk of pediatric-onset inflammatory bowel disease in offspring.

|  | **Low (n=54827)** | **High (n=5420)** |
| --- | --- | --- |
| Inflammatory bowel disease |  |  |
| Cases, n (%) | 158 (0.29%) | 10 (0.18%) |
| Unadjusted HR (95% CI)^2^ | Reference | 0.65 (0.34-1.24) |
| Adjusted HR (95% CI)^3^ | Reference | 0.68 (0.36-1.29) |
| Crohn’s disease |  |  |
| Cases, n (%) | NA^*^ | NA^*^ |
| Unadjusted HR (95% CI)^2^ | Reference | 0.70 (0.30-1.59) |
| Adjusted HR (95% CI)^3^ | Reference | 0.74 (0.32-1.70) |
| Ulcerative colitis |  |  |
| Cases, n (%) | NA^*^ | NA^*^ |
| Unadjusted HR (95% CI)^2^ | Reference | 0.60 (0.22-1.64) |
| Adjusted HR (95% CI)^3^ | Reference | 0.61 (0.22-1.67) |

^1^Maternal intake of organic meat was modeled as a binary variable (low versus high frequency of consumption) by combining responses ‘never’ and ‘sometimes’ as well as ‘frequently’ and ‘always’, respectively; ^2^Cox proportional hazards model; ^3^Cox proportional hazards model adjusted for maternal educational level, diet quality during pregnancy, pre-pregnancy body mass index, smoking during pregnancy, nutritional supplement use during pregnancy, antibiotics use during pregnancy, and parental inflammatory bowel disease diagnosis. *Numbers not presented to comply with General Data Protection Regulations and protect individual privacy. Abbreviations: CI, confidence interval; HR, hazard ratio.

# Supplementary Table 7. Associations of maternal organic fruit consumption^1^ during pregnancy with the risk of pediatric-onset inflammatory bowel disease in offspring.

|  | **Low (n=51986)** | **High (n=8261)** |
| --- | --- | --- |
| Inflammatory bowel disease |  |  |
| Cases, n (%) | 148 (0.28%) | 20 (0.24%) |
| Unadjusted HR (95% CI)^2^ | Reference | 0.86 (0.54-1.38) |
| Adjusted HR (95% CI)^3^ | Reference | 0.89 (0.55-1.43) |
| Crohn’s disease |  |  |
| Cases, n (%) | 85 (0.16%) | 10 (0.12%) |
| Unadjusted HR (95% CI)^2^ | Reference | 0.75 (0.39-1.45) |
| Adjusted HR (95% CI)^3^ | Reference | 0.79 (0.41-1.54) |
| Ulcerative colitis |  |  |
| Cases, n (%) | 63 (0.12%) | 10 (0.12%) |
| Unadjusted HR (95% CI)^2^ | Reference | 1.01 (0.52-1.97) |
| Adjusted HR (95% CI)^3^ | Reference | 1.02 (0.52-2.00) |

^1^Maternal intake of organic fruit was modeled as a binary variable (low versus high frequency of consumption) by combining responses ‘never’ and ‘sometimes’ as well as ‘frequently’ and ‘always’, respectively; ^2^Cox proportional hazards model; ^3^Cox proportional hazards model adjusted for maternal educational level, diet quality during pregnancy, pre-pregnancy body mass index, smoking during pregnancy, nutritional supplement use during pregnancy, antibiotics use during pregnancy, and parental inflammatory bowel disease diagnosis. Abbreviations: CI, confidence interval; HR, hazard ratio.

# Supplementary Table 8. Associations of maternal organic vegetable consumption^1^ during pregnancy with the risk of pediatric-onset inflammatory bowel disease in offspring.

|  | **Low (n=45830)** | **High (n=14417)** |
| --- | --- | --- |
| Inflammatory bowel disease |  |  |
| Cases, n (%) | 138 (0.30%) | 30 (0.21%) |
| Unadjusted HR (95% CI)^2^ | Reference | 0.70 (0.47-1.04) |
| Adjusted HR (95% CI)^3^ | Reference | 0.72 (0.48-1.07) |
| Crohn’s disease |  |  |
| Cases, n (%) | 78 (0.17%) | 17 (0.12%) |
| Unadjusted HR (95% CI)^2^ | Reference | 0.70 (0.42-1.19) |
| Adjusted HR (95% CI)^3^ | Reference | 0.72 (0.42-1.24) |
| Ulcerative colitis |  |  |
| Cases, n (%) | 60 (0.13%) | 13 (0.09%) |
| Unadjusted HR (95% CI)^2^ | Reference | 0.70 (0.38-1.28) |
| Adjusted HR (95% CI)^3^ | Reference | 0.71 (0.38-1.31) |

^1^Maternal intake of organic vegetables was modeled as a binary variable (low versus high frequency of consumption) by combining responses ‘never’ and ‘sometimes’ as well as ‘frequently’ and ‘always’, respectively; ^2^Cox proportional hazards model; ^3^Cox proportional hazards model adjusted for maternal educational level, diet quality during pregnancy, pre-pregnancy body mass index, smoking during pregnancy, nutritional supplement use during pregnancy, antibiotics use during pregnancy, and parental inflammatory bowel disease diagnosis. Abbreviations: CI, confidence interval; HR, hazard ratio.

# Supplementary Table 9. Associations of maternal organic cereal consumption^1^ during pregnancy with the risk of pediatric-onset inflammatory bowel disease in offspring.

|  | **Low (n=44642)** | **High (n=15605)** |
| --- | --- | --- |
| Inflammatory bowel disease |  |  |
| Cases, n (%) | 136 (0.30%) | 32 (0.21%) |
| Unadjusted HR (95% CI)^2^ | Reference | 0.68 (0.47-1.01) |
| Adjusted HR (95% CI)^3^ | Reference | 0.70 (0.47-1.05) |
| Crohn’s disease |  |  |
| Cases, n (%) | 78 (0.17%) | 17 (0.11%) |
| Unadjusted HR (95% CI)^2^ | Reference | 0.63 (0.37-1.07) |
| Adjusted HR (95% CI)^3^ | Reference | 0.65 (0.38-1.12) |
| Ulcerative colitis |  |  |
| Cases, n (%) | 58 (0.13%) | 15 (0.10%) |
| Unadjusted HR (95% CI)^2^ | Reference | 0.75 (0.43-1.33) |
| Adjusted HR (95% CI)^3^ | Reference | 0.78 (0.43-1.40) |

^1^Maternal intake of organic cereal was modeled as a binary variable (low versus high frequency of consumption) by combining responses ‘never’ and ‘sometimes’ as well as ‘frequently’ and ‘always’, respectively; ^2^Cox proportional hazards model; ^3^Cox proportional hazards model adjusted for maternal educational level, diet quality during pregnancy, pre-pregnancy body mass index, smoking during pregnancy, nutritional supplement use during pregnancy, antibiotics use during pregnancy, and parental inflammatory bowel disease diagnosis. Abbreviations: CI, confidence interval; HR, hazard ratio.

**References**

(1) Bjerregaard AA, Halldorsson TI, Tetens I, Olsen SF. Mother's dietary quality during pregnancy and offspring's dietary quality in adolescence: Follow-up from a national birth cohort study of 19,582 mother-offspring pairs. PLoS Med 2019;16(9):e1002911.

(2) Ministry of Food, Agriculture and Fisheries of Denmark. The Official Dietary Guidelines. Glostrup, Denmark: The Danish Veterinary and Food Administration, 2021. <https://foedevarestyrelsen.dk/Media/638198073401130807/Danish_Official_Dietary_Guidelines_Good_for_Health_and_climate_2021_SCRE.pdf>

(3) Tetens I, Pedersen AN, Schwab U, Fogelholm M, Þórsdóttir I, Gunnarsdóttir I, Andersen SA, Möltzer HM. Nordic Nutrition Recommendations 2012: Integrating nutrition and physical activity. Nordic Council of Ministers, 2014.
